# A radiation therapist’s guide to health literacy: A narrative review

**DOI:** 10.1002/jmrs.520

**Published:** 2021-06-16

**Authors:** Toni Kelly, Yolanda Surjan, Marianne Rinks, Helen Warren‐Forward

**Affiliations:** ^1^ School of Health Sciences University of Newcastle Australia; ^2^ Illawarra Shoalhaven Local Health District Australia

**Keywords:** barriers, health literacy, patient‐centred care, radiation therapist, screening

## Abstract

Radiation therapist (RT) communication plays an essential part of patient‐centred care in achieving better patient outcomes within radiation oncology. Patients present from a range of social circumstances, education levels and cultural backgrounds, all of which may significantly impact their level of health literacy (HL). Using literature sourced from databases such as EMCare Nursing & Allied Health Database, MEDLINE(R) and APA PsycInfo, this narrative review explores HL definitions, international comparison rates and indications of individual low HL. It also reviews HL assessments as well as exploring enablers and barriers to HL from the RT perspective. Strategies from both the individual or organisational perspective are provided for RTs to begin or continue their HL interest. By educating the radiation therapy profession about health literacy and making small changes in interpersonal interactions, there is the opportunity to impact patients’ experiences and outcomes significantly.

## Graphical abstract

Radiation therapist (RT) communication plays an essential part in patient‐centred care in achieving better patient outcomes within radiation oncology. This article explores a range of definitions, international comparison rates and strategies on health literacy from the RT perspective. By educating the radiation therapy profession about health literacy, making small changes in interpersonal interactions, there is the opportunity to make a significant impact on patients’ experiences and outcomes.

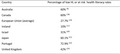

## Introduction

Patient‐centred care has, over the last two decades, become internationally recognised as imperative to high‐quality health care.^1^ A customised approach for care considering unique patient needs, concerns and preferences may lead to more effective and productive health outcomes. The eight dimensions of patient‐centred care encompass family and friends' involvement, care coordination, respect for patients’ preferences, physical comfort, access to care, continuity and transition, emotional support, information, communication and education.^2^ Within the radiation therapy environment, the patient must understand their diagnosis and treatment to the best of their ability. For radiation therapists (RTs), the understanding and knowledge of health literacy (HL), as a mechanism to assist in this process, is necessary to deliver patient‐centred care. This narrative review aims to offer insight into the definition, environment, screening tools, enablers and barriers of HL relevance for RTs and provide recommended strategies for improved patient outcomes.

## Methods

### Search Strategy

To identify relevant articles, the following electronic databases were searched: EMCare Nursing & Allied Health, MEDLINE(R) All including Epub Ahead of Print, In‐Process & Other Non‐Indexed Citations, Daily and Versions(R) and APA PsycInfo. Combinations of the following search terms (keyword and MeSH) were used: ‘health literacy or health literacy.mp’. ‘health knowledge’, ‘attitudes’, ‘practice’, ‘strategy’, ‘barrier’, ‘communication barriers’, ‘barrier.mp.’ ‘radiation therapy.mp.’, ‘radiotherapy’, ‘patient education’, ‘patient outcome assessment’, ‘assessment.mp.’, ‘screen’. The search strategy had limits set for humans and the English language. Reference lists of all retrieved papers were manually searched to identify any articles not located by the electronic search.

### Study selection

Information provided in the title, abstract and keywords was assessed to make a decision about the article’s suitability for inclusion. Where there was insufficient information in the title and abstract to determine suitability for inclusion, the full paper was retrieved and reviewed.

## Results

The results of the literature search were divided into six themes:

- Health literacy definitions
- Individual health literacy
- Health literacy environment
- Health literacy screening and assessment tools
- Enablers and barriers of health literacy
- Health literacy and the role of the radiation therapist

### Health literacy definitions

In the 1990s, HL focussed on reading literacy and patient comprehension.^3^ It is now commonly separated into two components; individual HL and the HL environment.

Individual HL is defined as the skills, knowledge and capacity of an individual to understand, evaluate and follow health information or advice.^4^ Individuals with low HL may misunderstand information relating to disease and disease processes, treatment options and healthcare navigation, leading to poorer healthcare outcomes.^4^ Cancer patients with low HL may have trouble understanding bladder and bowel preparation instructions necessary for some radiotherapy treatments and the implications of non‐compliance. Outcomes from non‐compliance result in repeated bladder scanning and imaging before treatment, adding extra time to their appointment and extra radiation dose from unnecessary repeated imaging.

The HL environment is the infrastructure, policies, procedures and employees working within the health system. Each of these has the potential to impact how health‐related information is understood and accessed by individuals.^4^ While these aspects of the HL environment could be categorised as organisational, researchers have further evolved the HL model^5^ to provide a health care professional (HCP) hierarchical definition^6^ and patient perspective model of HL^7^ (Table 1). Zarcadoolas et al. characterised a multi‐dimensional HL model featuring four central domains; fundamental, science, civic and cultural HL.^5^ In comparison, Nutbeam expressed a hierarchy of HL, beginning with functional, moving to communicative, with the highest achievable HL being critical literacy skills.^6^ Jordan et al. identified HL from the patient perspective identifying seven HL abilities,^7^ whereas Edwards et al. developed a five‐stage model demonstrating an HL trajectory.^8^ Guzys^9^ argues that public health literature places considerable importance on the concept of critical HL. However, the focus still sits on assessing the individual instead of evaluating HL at the societal level to provide better health outcomes.^9^

**Table 1:** Summary of health literacy models.

Author	Health Literacy Model	Model features
Zarcadoolas et al.^5^	Multi‐dimensional model divided into four central domains	Fundamental health literacy (skills and strategies required for reading, writing and numeracy Science literacy (competence in interpreting science and technology‐associated concepts) Civic literacy (awareness and knowledge of public issues, government issues and their impact on public health) Cultural literacy (ability to recognise customs, social identity world views to interpret and make decisions around health information)
Nutbeam^6^	Expression of hierarchy of health literacy	Functional (basic level including reading and writing) Communicative literacy (comprising of advanced cognitive and literacy skills) Critical literacy skills (skills that one uses to exert better control of social identity)
Jordan et al.^7^	Health literacy from the patient perspective Seven abilities identified	Knowing when to and where to seek health information Verbal communication Assertiveness Literacy Ability to retain information Ability to process information Application of skills
Edwards et al.^8^	Five stage model demonstrating health literacy trajectory over time	Stage One identified basic health knowledge Stage Two is developing health literacy skills and practices Stage Three demonstrates health interactions citing active involvement in discussion with Health Care Professionals Stage Four uses informed options from stage three to assist with independent decision‐making Stage Five is making informed decisions where considerations of all options have taken place
Guzys et al.^9^	Advocate for Public/societal health literacy on critical health literacy	Advocates evaluating the health literacy at the societal level within the community to provide better health outcomes, instead of society’s focus on assessing the individual

Health literacy definitions within radiation oncology^10^, ^11^, ^12^ align with the Nutbeam^6^, ^13^ model, acknowledging a hierarchy of HL. While relevant, when teaching HL theory and skills to RTs, Kelly et al.^14^, ^15^ suggest a more organisational approach to HL where both the individual HL and HL environment are acknowledged.

### Individual health literacy

HL is currently an internationally recognised issue with large international individual HL disparities.^16^ Table 2 provides examples of global variations of individual low HL. Australia’s low HL rate of approximately 60% according to the Adult and Life Skills Survey (ALLS) measured by the Australian Bureau of Statistics (ABS) in the 2006 census results in a large proportion of the population lacking an understanding of a modern healthcare system's complex demands.^4^ In 2018, the ABS collected data through the National Health Survey, known as the Health Literacy Questionnaire (HLQ) ^17^ where 44 items were classified into nine domains (Table 3). The Australian government recognises that outcomes from the HLQ and ALLS are different and cannot be compared directly.^17^ Similarly, care is needed when comparing Australian HL results with international low HL statistics due to differing methodologies and timeframe differentials. Therefore, The Netherlands reports high levels of HL with 25% of the population demonstrating an excellent level of HL and 1.8% indicating inadequate or low HL.^24^ Similarly, Ireland’s population recorded 21% demonstrating excellent HL and 10% with inadequate or low HL.^25^ International HL variation is difficult to quantify; however, Mantwill et al. suggest racial/ethnic disparities are acting as a proxy for predictors of health disparities.^26^ Australia maintains a high migrant population with 26.3%^27^ of people born overseas, many of which have English as a second language. The Netherlands has only 4.75% of foreign nationals in their population,^24^ and Ireland has 12.7%,^25^ both much lower rates than Australia.

**Table 2:** Comparison of international low or at‐risk health literacy populations.

Country	Percentage of low HL or at‐risk health literacy rates
Australia	60% ^4^
Canada	60% ^18^
European Union (average)	27.7% ^19^
Ireland	10% ^11^
Israel	31% ^20^
Japan	60.1% ^21^
Portugal	72.9% ^22^
United Kingdom	42% ^23^

**Table 3:** Domains of Australian health literacy questionnaire.^17^

Domain 1:	Feeling understood and supported by healthcare providers
Domain 2:	Having sufficient information to manage my health
Domain 3:	Actively managing my health
Domain 4:	Social support for health
Domain 5:	Appraisal of health information
Domain 6:	Ability to actively engage with healthcare providers
Domain 7:	Navigating the healthcare system
Domain 8:	Ability to find good health information
Domain 9:	Understand health information well enough to know what to do

The World Health Organisation recommends competencies that validate an individual to be classified as ‘health literate’^19^ (Table 4). These competencies are categorised into four dimensions of health information processing: the ability to access, understand, process and apply all domains of health care, disease prevention and health promotion.^19^ Expectations that individuals demonstrate HL over such broad topic areas could arguably be deemed as ambitious. For example, individuals employed in health, such as nurses diagnosed with cancer, do not necessarily have radiation therapy HL and therefore may not understand radiation therapy associated concepts and jargon.

**Table 4:** Demonstrated abilities of a health literate citizen.

	Access	Understand	Process	Apply
Health Care	Ability to access al information on medical or clinical issues	Ability to understand medical information and derive meaning	Ability to interpret and evaluate medical information	Ability to make informed decisions on medical issues
Disease Prevention	Ability to access information on risk factors for health	Ability to understand information on risk factors for health and derive meaning	Ability to interpret information on risk factors for health	Ability to make informed decisions on risk factors for health
Health Promotion	Ability to update oneself on determinants of health in the social and physical environment	Ability to understand information on determinants of health in the social and physical environment and derive meaning	Ability to interpret and evaluate information on health determinants in the social and physical environment	Ability to make decisions on health determinants in the social and physical environment

Reproduced with permission from: Sorensen K et al.^19^

#### Indicators of limited Individual Health Literacy

Patients may use a variety of methods in an attempt to hide their low HL. Montgomery^28^ and Quinn et al.^11^ propose examples of concealment that include incomplete form filling, inability to explain the purpose of treatment, missed appointments and demonstrating avoiding body language when asked to read something.^29^ Smith et al.^12^ suggest that verbal cues including general language ability, absence of questions, issues with comprehension and limited responses to questions also indicate low HL. Non‐verbal cues that may alert RTs of a low HL status can include negative facial expressions, inability to self‐care and inability to tolerate new information.^11^ Proxies for HL difficulties such as socio‐demographic features, residing in a remote location, lower socio‐demographics, level of education, ethnicity, non‐English speaking background (NESB) or English as a second language (ESL) and current level of employment can also alert to lower HL.^12^ Quinn et al.^11^ add that the use of illegal drugs, alcohol overuse and older age may also impact HL status.

### Health literacy environment

Approaches to organisational HL require a solid and committed leadership approach, with managers modelling what HL should look like within their organisation.^30^ It is essential to empower all individuals within the organisation to play their role. Significant pre‐planning and upskilling of HCPs with knowledge of HL and a commitment to improving all aspects of HL within the organisation are essential. Empowering RTs to begin or continue the conversation around organisational HL within their facility using published resources may improve the patient experience. To enhance organisational HL, the United States Institute of Medicine^30^ has published ten attributes that define a health literate organisation (Table 5). To embed such features, it is essential to generate stakeholder ownership. Periodic collaboration, including monthly meetings with stakeholders and communication of meeting action items to all staff, will also improve HL.

**Table 5:** The ten attributes of health literate organisations.

	Dimensions of a Health Literate Organisation
1.	Has leadership that makes health literacy integral to its mission, structure and operations.
2.	Integrates health literacy into planning, evaluation measures, patient safety and quality improvement.
3.	Prepares the workforce to be health literate and monitors progress.
4.	Includes populations served in the design, implementation and evaluation of health information and services.
5.	Meets the needs of populations with a range of health literacy skills while avoiding stigmatisation.
6.	Uses health literacy strategies in interpersonal communications and confirms understanding at all points of contact.
7.	Provides easy access to health information and services and navigation assistance.
8.	Designs and distributes print, audio‐visual and social media content that is easy to understand and act on.
9.	Addresses health literacy in high‐risk situations, including care transitions and communications about medicines.
10.	Communicates clearly what health plans cover and what individuals will have to pay for services.

Reproduced with permission from: Brach C et al.^30^

### Health literacy screening and assessment tools

HL screening involves the assessment of an individual's level of HL. Comparisons of various tools in a range of settings help inform efficiency and efficacy when performing HL assessments.^31^, ^32^, ^33^

As noted by Moore,^34^ tests or screening tools are distributed into four main classifications: word recognition tests, reading comprehension tests, functional HL tests and more informal methods. The focus within this narrative will be on functional and informal HL testing.

#### Functional HL

Functional HL is the ability to demonstrate comprehension and act upon health information.^33^ The Test of Functional Health Literacy in Adults aims to measure patient comprehension by interpreting information on a prescription label categorising results as inadequate, marginal or adequate.^35^ Another HL assessment tool, the Newest Vital Sign, assesses HL using a nutrition label and individuals' ability to answer six questions within three minutes.^36^ The Cancer Message Literacy Test‐reading (CMLT‐r) and CMLT‐listening help measure comprehension of written and spoken cancer prevention and screening information, respectively.^37^ While these tests have a place to determine a patient’s HL level, there is a reluctance to use any official testing in radiation oncology^10^, ^11^, ^12^, ^38^. Williams et al.^38^ state that *‘radiation oncology departments are not suitable to sit literacy tests as patients are dealing with a cancer diagnosis, and alternative informal methods for identifying HL in this setting are required’* ^38^pp. S12.

#### Informal tests

Informal tests involve observing patient behaviours, including not completing forms or requesting help completing forms where the reason is they have forgotten spectacles.^34^ Examples within radiotherapy include patients with low HL missing scheduled treatments as they may not fully comprehend the treatment requirements and patients not adhering to recommended skincare guidelines due to a lack of understanding, such as wearing makeup in the treatment region.

### Emotions surrounding HL screening

Shame, anxiety and embarrassment are concealed emotions associated with low HL.^39^ The burden of low HL can stigmatise patients, contributing to feeling fearful. Parikh et al.^39^ report from their study in Atlanta, Georgia, that 39.7% of 202 patients identified as having low functional HL also admitted feelings of shame. Parikh suggests HCPs should be informed of the problems of low HL within their particular setting, including those identified through clinic registration processes (such as providing demographic details), understanding of verbal information and written materials and be acutely aware of the possibility of shame and embarrassment this group of patients may have.

Farrell et al.^40^ using qualitative semi‐structured interviews from an eleven patient study within a primary care setting, in the United States, report phrases such as ‘how often’ and the word 'help' were acknowledged as potentially embarrassing. Screening was preferred in a private room, where 73% of participants felt the patient was responsible to initiate a discussion about HL.^40^

Jordan et al.^7^ suggest that most HL assessments are performed as the HCP evaluates the patient; however, there should be a consideration from the patient perspective. Using Patient‐Reported Outcomes (PROs) to ask patients about their abilities, identifying specific patient needs and customising information based on answers would fit a patient‐centred care approach more closely.^7^ PROs can reduce patient shame and embarrassment often associated with HL testing.^39^ Conversely, HCP's education around low HL would be sensitive to patient’s needs, reducing the stigma associated with low HL. Within the radiotherapy setting, Quinn et al.^11^ interviewed 16 RTs across Ireland and reported most RTs felt there was no shame associated with low HL within radiation therapy patients generally; however, documenting in the patient notes could induce some level of embarrassment.^11^ If it is determined the patient is part of the ‘hidden population’ with low HL, RTs must constantly, but discreetly, check the patient understanding without inciting shame or embarrassment.^11^

### Enablers and barriers of health literacy

Edwards et al. suggest the *‘communication styles of HCPs either facilitate information exchange and enable empowerment or sometimes act as a barrier to information exchange and disempower patients’*.^8^ ^p2^ Paradoxically, the HCP becomes the hinge point based on the assessment of the patient's level of HL; if accurate, they will provide information at a rate the patient can understand by checking in during the information giving process. If overestimated, they may further confuse the patient. HCP’s must know this critical point for awareness, understanding and ability to act in the provision of patient‐centred care.

#### Enablers

Edwards et al.^8^ affirm several points to enhance the patient experience, including personal motivation, emotion management and involving family within consultations. Patients who can seek information on the Internet may understand their diagnosis from an emotional perspective, reducing anxiety.^8^ Involving family to act as HL interpreters provides a support mechanism for patients.^8^ Hughson et al.^41^ suggest utilising technology to improve the patient experience, including adopting smartphone applications to reduce the HL gap and hospital developed resources (with the patient and carer access) can contribute to positive HL patient experiences.^41^

#### Barriers

Barriers to HL are categorised into patient barriers, HCP barriers and organisational barriers.

##### *Patient* *barriers*

Edwards et al.^8^ propose patient barriers may include decreased motivation, rejection of diagnosis, general attitudes towards one's health and reduced help‐seeking behaviour. Emotional barriers (shock, fear and anxiety) can prevent connecting with information provided, and within a cancer setting, this is not unusual. Unpleasant memories from a family or friend receiving a similar diagnosis can surface providing a barrier to future information processing. Finally, the embarrassment of the stage of presentation (of the malignancy) can also present as a patient barrier to HL.^43^

Hughson et al.^41^ further categorise patient barriers into cultural and social domains, citing pressures from family, authority figures and patient access to services. Patient no‐shows occur as lower importance is placed on the appointment.^41^ Contextually, there can be major implications for a ‘no‐show’ patient. An interruption to treatment, including missing scheduled treatments, can result in a loss of tumour control, particularly if the tumour cells are rapidly repopulating.^42^ Future makeup sessions for missed days may be considered, including twice‐daily treatments or treatment on weekends to attempt to regain tumour control.^42^

From an indigenous viewpoint, Lambert et al.^43^ believe exclusion from western education has meant that the HCP identifies this fact as a barrier to building HL. Furthermore, Lambert stresses that limited western education of patients with a different cultural identity can lead to unfamiliarity with biomedical words causing misunderstanding of health information, or assumed low HL.^43^

Thompson et al.^44^ explored through a systematic review, the impacting factors affecting rural patients access to radiotherapy services. Distance, socioeconomic factors and carer support were cited as crucial decision‐making points or barriers to access when rural patients were considering radiotherapy.

##### *HCP* *barriers*

Time constraint is a common barrier reported by HCPs.^10^, ^11^, ^41^, ^45^, ^46^, ^47^ Time allocation for patient‐related tasks is generally standardised and usually developed over time using pre‐existing data. For example, completing a breast treatment takes typically 15 minutes. Acknowledging extra time should be scheduled to identify the HL status of the patient and then acting accordingly to ensure patient needs are met.

Other HCP barriers include poor communication skills undermining patient engagement opportunities and dismissing the patient's ideas or not offering more information.^8^ It can be confusing to patients when HCPs are not engaging in active listening or actively withholding information.^8^ HCPs should be recognising the HL trajectory stage (Edwards et al. study in Table 1) and customising patient information and encourage patients to seek out information themselves in a supportive manner.^8^ RTs ought to be recommending resources such as the Cancer Council for information regarding diagnosis and quality of life topic areas.

HCPs may impede information provision to patients in fear of appearing condescending to highly literate patients.^14^, ^46^, ^48^ Checking for patient understanding using the teach‐back method of asking the patient to repeat in their own words what they need to understand or do as part of the information provision process^4^ can be an issue for some HCPs.

##### Organisational barriers

Farmanova et al.^49^ identify pitfalls commonly made at an organisational level when implementing an HL strategy. Knowing these barriers ahead of organisational HL strategy implementation may help prepare effective organisational change within any facility, including radiotherapy departments. The pitfalls are divided into three categories; organisational and leadership, design and planning improvement interventions and human resources. Drawbacks within the first category include reprioritisation of health activities and none or minimal buy‐in from the leadership team. The design and improvement category's challenges include no change champions, a deficiency of policies and procedures supporting HL practice, and a lack of time and resources. The final category cites unclear staff roles, inadequate training and unawareness of HL.

### Health literacy and the role of the radiation therapist

Low HL is generally associated with poor outcomes for at‐risk patients.^3^, ^50^, ^51^ This is particularly significant within radiation therapy^12^, ^28^, ^38^ where an increased risk of patient morbidity may arise due to poor adherence to treatment regimens as a result of reduced understanding.

When overwhelmed by anxiety or worry, it may prove inevitable that individuals may be at risk of low HL, particularly when hearing new or distressing information.^52^ Effective HCP communication has the power to diminish worry or anxiety, and this can assist with improving outcomes during a difficult time.^53^ Therefore, RTs need to provide clear, concise education to patients about their treatment across a range of common issues, such as side effects, skincare and nutrition.^28^

RT awareness of HL and low HL is still a significant issue.^11^, ^15^, ^18^, ^28^ Communication skills training inclusive of HL statistics, strategies and practice is paramount to reduce the gap of patients misunderstanding of their treatment.^15^

#### Effective health literacy strategies for radiation therapists

Smith et al.^12^ present strategies to be used by RTs to manage and respond to patients of different HL statuses, to deliver adequate/ clear information and reduce patient anxiety (Table 6). These strategies are categorised into three activities; timing of information, tailoring information to match the HL level and enhancing understanding. This table does come with a caveat that RTs must have a perspective of the patient’s HL level prior to enacting these strategies. Strategies are then categorised into the RTs perceived levels of the patient HL to promote the most efficient interaction.

**Table 6:** Strategies used to manage and respond to patients of all levels of health literacy by radiation therapists.

Activity	Strategies used by radiation therapists
Timing of information	*Irrespective of health literacy* Treatment planning and first day of treatment‐key time points where RT provided a basic outline of what treatment entails. Staggering information provision as treatment progresses. Verify understanding and ‘filling’ the missing knowledge, or refer to another member of the cancer team to explain. Reiterating information as information provided at the beginning may have been lost or forgotten as treatment progresses.
Tailoring communication to match health literacy level	*Irrespective of health literacy* At the beginning of treatment, information pitched at a basic level Communication and language tailored to health literacy level in accordance with verbal and non‐verbal cues. Preference for verbal communication, but reinforced by visuals, written and audio‐visual information. Use of visual prompts (eg model of radiotherapy mask, photographs of CT machine or models of machinery in treatment room) Provision of information produced by the radiation oncology department (DVDs., written booklet/brochure) Risk communication preference for qualitative descriptors (eg very unlikely) over numbers, more comfortable discussing side effects than issue relating to efficacy of treatment *Patients perceived to have lower health literacy* Greater use of plain/lay language Minimal use of medical terminology Convey the basic facts with minimal scientific or technical information Greater use of analogies to convey complex concepts (eg ‘taking an x‐ray is like taking a picture’) *Patient perceived to have higher health literacy* Provide information about the technical aspects of radiotherapy Addressing ‘why’ rather than ‘what’ questions Greater use of medical terminology/concepts, but avoided using highly specialised technical terms. Talking to them ‘at their level’ and ‘not talking down’
Enhancing understanding	Repetition and asking the person to repeat back to the radiation therapist what they have understood in their own words. Encouraging question asking Reminders and prompts regarding scheduled appointments, medication and self‐care. Check up on information processing throughout treatment and reinforcing the essential aspects Inviting family members or support person with higher literacy to attend the consultation to help reinforce information to the patient Going through written information with the patient and checking understanding

Reproduced with permission from: Smith SK et al. ^12^

Williams et al.^38^ offer communication strategies specifically for RTs when managing low HL patients, beginning with speaking in plain English. It is essential to ensure contextual radiation therapy jargon is translated into language a 12‐ to 14‐year‐old could understand.^4^ Kelly et al. suggest some options; ‘erythema’ can be explained as a reddening of the skin, ‘toxicities’ can be described as side effects that make you sick and ‘bolus’ is material added to the skin to trick the radiation into depositing the dose closer to the skin surface.^15^ A worthwhile exercise is an RT group session to arrive at more straightforward explanations for more commonly used jargon that may require additional explanation to patients.

The teach‐back method to check for understanding for information patients need to remember or retain is also suggested.^4^ Two examples of phrasing include ‘*so I can be sure I have explained your first treatment session information clearly, could you please tell me what you need to remember?’* and ‘*before you go, I want you now to tell me what are the main instructions to prepare your bladder for treatment each day, so I can be sure I have explained them correctly?’* ^15^ pp. 221.

The *‘Chunk and Check’* method recommended by the Health Literacy Place^54^ suggests breaking down information into manageable ‘chunks’ when presenting lots of information, such as during pre‐simulation or pre‐treatment patient education sessions. Providing two to three points of information before checking for understanding is recommended. For example, providing patients who require their bladder full for treatment, the volume of water and timing to wait before treatment are two points that should be easily remembered.

Using open‐ended questions (e.g. ‘*what questions do you have?’*) and limiting the use of closed questions (yes/no answers) during information provision sessions is also advocated.^38^ For example, when asking a closed question, ‘Do you have any questions?’ requiring a yes/no response is not encouraging clarification.^38^ Montgomery^28^ makes similar suggestions; avoiding jargon, speak slowly and consider other media such as pictures, video and computer presentations. Avoiding anatomical words, using an active voice, utilising interpreters when available, demonstrating positive body language and creating a shame‐ and blame‐free environment if assessing HL.^27^

## Conclusion

This article has explored HL from an RT perspective to assist with understanding the impact of low HL within the profession. Knowledge of low HL international statistics and HL barrier awareness is the beginning to improving patient outcomes. Identifying patient indicators of low HL and implementing known HL techniques are small manageable daily changes to promote patient‐centred care. From an organisation viewpoint, evaluation and assessment of current HL policy need to be identified by managers and HL champions motivated to drive and create practice change. Changes, when executed well, endeavour to improve the entire patient experience.

## Conflict of Interest

The author declares no conflict of interest.
